# Does Orthokeratology Wearing Affect the Tear Quality of Children?

**DOI:** 10.3389/fped.2021.773484

**Published:** 2022-01-18

**Authors:** Zhengyang Tao, Jiao Wang, Minjuan Zhu, Zhihong Lin, Jun Zhao, Yu Tang, Hongwei Deng

**Affiliations:** ^1^The Second Clinical Medical College, Jinan University, Shenzhen, China; ^2^Department of Strabismus and Pediatric Ophthalmology, Shenzhen Eye Hospital, TheClinical Medical College of Shenzhen University, Shenzhen, China; ^3^Department of Strabismus and Pediatric Ophthalmology, Shenzhen Eye Hospital Affiliated to Jinan University, The Second Clinical Medical College of Jinan University, Shenzhen, China; ^4^Department of Ophthalmology, The Second Clinical Medical College of Jinan University, Longhua Branch Institute of Shenzhen People's Hospital, Shenzhen, China; ^5^Department of Strabismus and Pediatric Ophthalmology, The Jinan University of Shenzhen Eye Hospital, Shenzhen University of Medicine, Shenzhen, China

**Keywords:** tear quality changing, orthokeratology wearing, children, Newcastle-Ottawa scale, pediatric ophthalmology

## Abstract

Orthokeratology is currently known as one of the most effective methods of myopia control in the process of rapid deterioration of the global myopia prevalence. As orthokeratology is widely used, it is necessary to evaluate its complications reasonably and accurately. Eye surface problems in children, such as dry eyes, have received increasing attention. At present, there is no conclusive evidence on how orthokeratology affects the ocular surface, especially the tears. To our knowledge, this is the first study to explore the relationship between orthokeratology lenses and tears through meta-analysis. However, it is still challenging to get a convincing conclusion and a higher level of evidence in this meta-analysis. Reasons for this include limitation of study design, lack of clarity on important confounding factors, lack of appropriate statistical tools, and other biases. This paper will analyze the dilemma existing in the current research from different perspectives to provide meaningful information for future studies in this field.

## Introduction

In the past 20 years, the number of people who developed myopia has increased by about 40% worldwide, mirroring a trend that will continue to rise sharply (1). In the face of this data, a large number of studies and achievements related to myopia control have emerged in recent years, such as studies on atropine eye drops and the progressive addition of spectacle lenses. Currently, orthokeratology is considered to be one of the most effective interventions for myopia control (2, 3).

**Figure 1:**
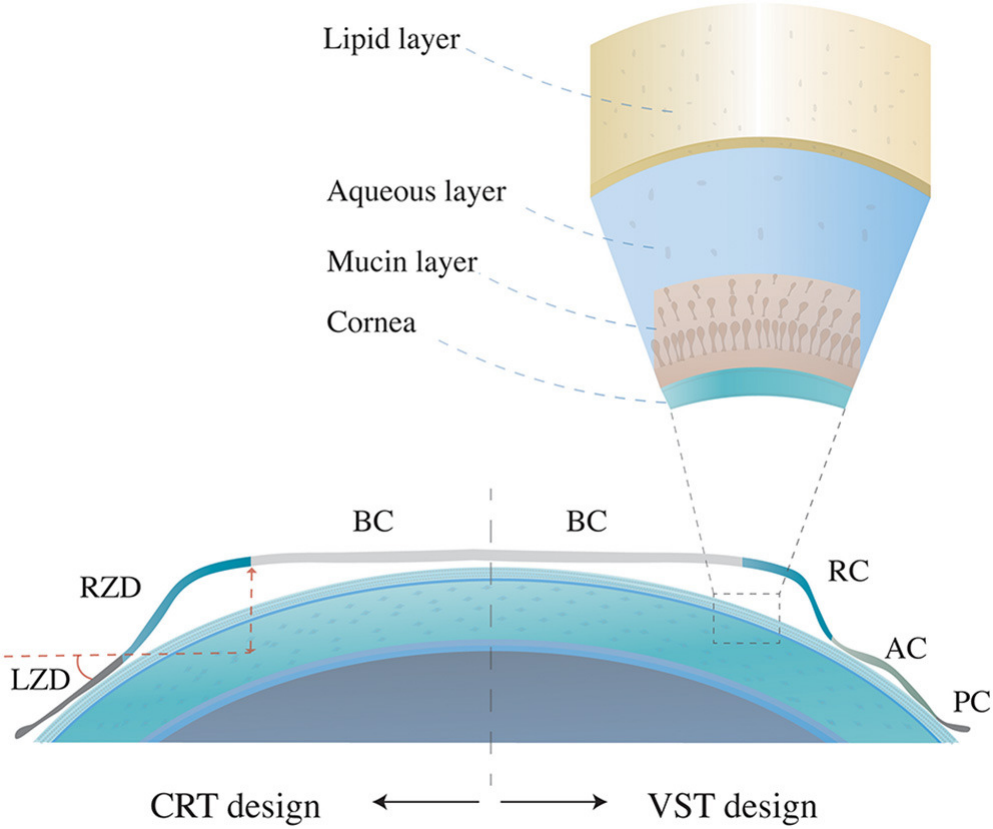
Orthokeratologylenses with different designs and the layers of tears.

There are two main design types of orthokeratology lenses, vision shaping treatment (VST) and corneal refractive therapy (CRT) (4). The VST design has four or more curves, including a base curve (BC) that compresses the cornea center, a reverse curve (RC) that causes the central tears to gather and thus promotes the flattening effect of the central lens on the corneal surface, an alignment curve (AC) for the correction of lens fixing and position, and a peripheral curve (PC) for tear exchange in the outermost circle (5). The CRT-designed orthokeratology lens is also widely used in the clinics, including three adjusted independent zones, the central BC, the return zone depth (RZD), and the outermost landing zone angle (LZA) (Figure 1). By wearing the reverse geometry lenses overnight, the thinned central corneal epithelium and the thickened peripheral corneal epithelium and stroma will build a satisfying visual acuity during the daytime. Current studies speculate that correction of central retinal vision and peripheral myopic defocusing effect may be the mechanism of orthokeratology to inhibit axial length growth (6).

The Dry Eye Workshop II defined dry eye disease as a multifactorial disease where the loss of homeostasis of the tear film has an etiological association with inflammation, damage, and neurosensory abnormalities (7). Dry eye syndrome has long been neglected in pediatric ophthalmology, for a clear correlation with age has been demonstrated in previous studies (8–10). However, the incidences of dry eye in children have received more attention in recent studies, which may be related to environmental changes and the increased use of electronics (11, 12).

As a myopia control method, mainly aimed at children and needing to be worn in direct contact with the ocular surface for a relatively long time, the orthokeratology lens has attracted many researchers' interest in how it affects the ocular surface (13–21). At present, the effect of orthokeratology on the ocular surface, especially on tears, which is one of the most critical components in maintaining the micro-ecology of the ocular surface and has not been definitively determined. In this study, the term “tear quality” refers to tear film stability and tear secretion. In order to explore the influence of orthokeratology wearing on tear quality, our team attempted to conduct a meta-analysis of the relevant literature on this topic, through which a conclusion with a higher level of evidence could be obtained. Additionally, the evidence level of the current literature would be analyzed in this process.

## The Meta-Analysis With A Confusing Result

### Methods

#### Search Strategy and Data Collection

The related literature was collected from the following databases: Pubmed, Embase, the Cochrane Library, and Chinese databases, including Wanfang and the China National Knowledge Infrastructure. The collected articles were published up to Oct 19, 2020. Search terms included the subject terms and random terms “orthokeratology lens” and “tear break-up time.” The detailed search strategies are found in Appendix 1. No language or time restrictions were adopted during the search.

#### Selection Criteria

The literature was managed through EndNote X9 software. The criteria of the articles included were: (1) prospective studies, (2) study content about the effect of orthokeratology lenses on the ocular surface, (3) leading observation indicators, including the average tear break-up time at baseline and at one, three, six, and twelve months follow-up (with repeated measurement at least three times, where a mean value and standard deviation represented the results), (4) subjects were between the ages of 6–18, (5) complete data. Studies would be removed if they were case reports, commentaries, reviews, letters, or conference abstracts. Zhengyang Tao and Zhihong Lin conducted the article-selection process. When the two authors had differing opinions on the inclusion or exclusion of a specific article, Hongwei Deng would act as arbitrator.

#### Quality Assessment

The quality of eight of the included articles was evaluated by the Newcastle–Ottawa scale (NOS). The counterpart of one article (a randomized controlled trial) was assessed with the Jadad scale. The total score on the NOS scale is 9 stars, with a maximum of 2 stars for the comparability column and a maximum of 1 star for the remaining ratings. A score from 5 to 9 on the NOS scale was considered relatively high quality. The scores of the Jadad scale ranged from 0 (very poor) to 7 (rigorous), in which maximum points for randomization, concealment of allocation, double-blind, and dropouts are two, two, two, and one, respectively. Jiao Wang and Minjuan Zhu conducted the grading process. When the two authors had differing opinions on the grading of a particular article, Jun Zhao would act as arbitrator.

#### Data Analysis

Statistical analyses were performed using Stata software (version 15.1) and R software for Windows (version: 3.5.2, Ross Ihaka and Robert Gentleman, University of Auckland, New Zealand). A Q test and I^2^ statistics were applied to evaluate the heterogeneity of the studies. When the heterogeneity was large, sensitivity analysis was used to further analyze the source of heterogeneity. Due to the small number of included studies, subgroup analysis will not be considered for use. The null hypothesis that the studies are homogeneous would be rejected if the *P* value was < 0.05 or the I^2^ was >50%. The total effect of the mean difference and 95% confidence interval (95% CI) were calculated using the random-effects model (DerSimonian–Laird, DL). The primary research indicator of this study was tear break-up time (TBUT), by comparing the TBUT value after orthokeratology wearing for 1 month and 6 months as the baseline, respectively. There were two secondary indicators, the tear secretion (through a Schirmer I test) and the Ocular Surface Disease Index (OSDI), comparing the two values after orthokeratology wearing for 6 months as the baseline.

### Results

#### Included Studies and Data Characteristics

**Figure 2:**
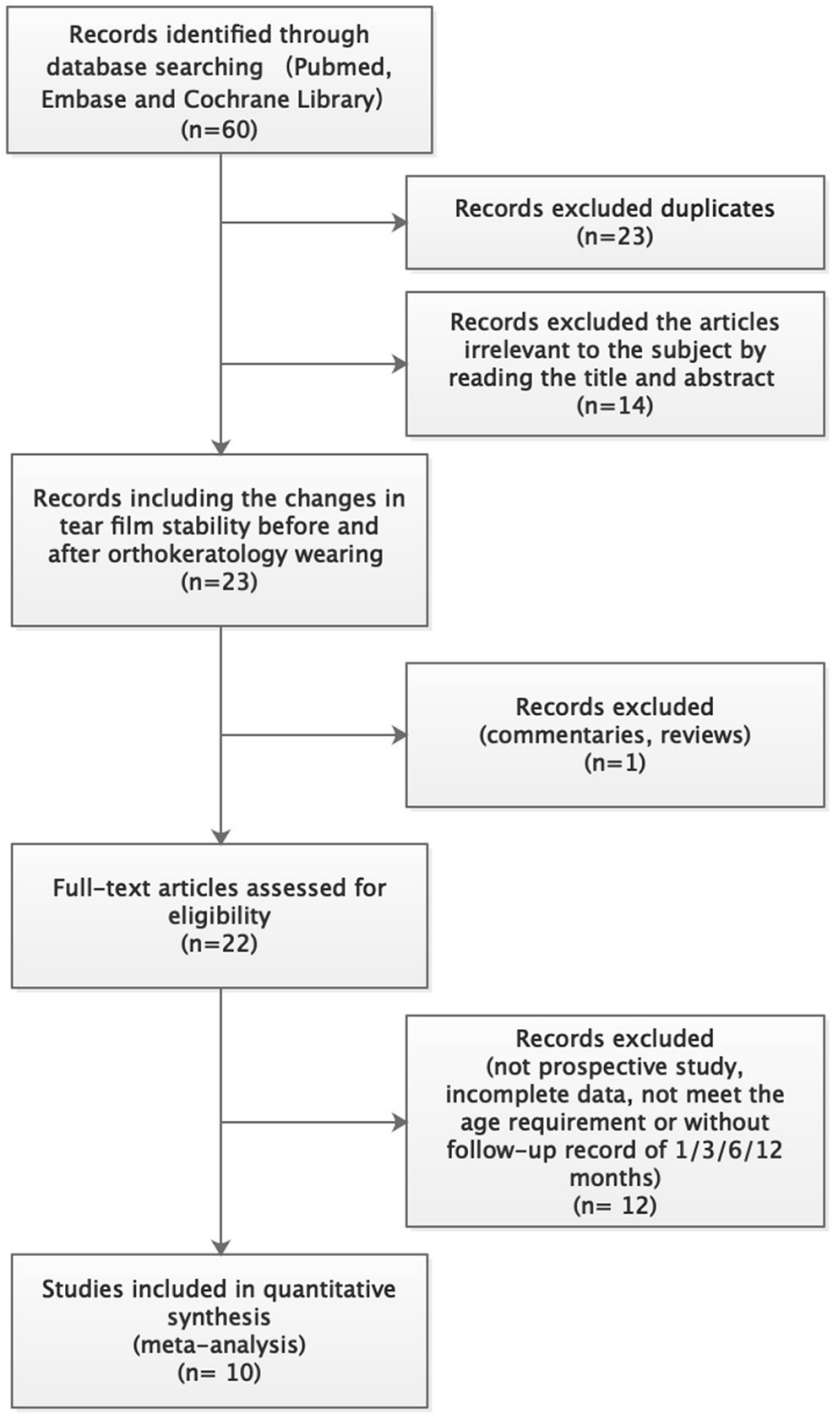
The detailed literature screening process.

A systematic literature search obtained 60 articles. Among them, 23 articles were excluded due to duplication. By reading the titles and abstracts, 14 articles unrelated to this study were excluded. One review and 12 studies with insufficient follow-up were also excluded. Finally, 10 articles were included in this meta-analysis (13–21). The detailed literature screening process is shown in Figure 2. The characteristic information of the 10 included literatures is shown in Table 1.

**Table 1:** Characteristic of the included studies in the meta-analysis.

**ID**	**Author (Years)**	**Type of study**	**Country**	**Recruiting period**	**Follow-up, Months (recorded time point)**	**Number of patients (eyes)**	**Gender (M/F)**	**Age (Mean ±SD) (Range)**	**Spherical equivalent (Mean ±SD)**	**Sleeping duration**	**Cleaning the lens care solution before wearing**
1	Li et al. (13)	Prospective, one-arm study	China	2013.01–2013.08	12 (1,3,6,12)	40 (80)	22/18	13.68 ± 2.32 (11–18)	−3.61 ± 1.48	8–10 h	NA
2	Liu et al. (14)	Cohort study	China	2017.03–2018.05	12 (1,3,6,12)	36 (72)	15/21	13.52 ± 1.12 (12–18)	−2.94 ± 1.30	8–10 h	NA
3	Na et al. (15)	Prospective, one-arm study	South Korea	NA (study period: 2010.01–2014.07)	36 (6,12,24,36)	58 (58)	18/40	NA (7–18)	−2.97 ± 2.02	8 h	NA
4	Shi et al. (16)	Cohort study	China	2013.10–2013.12	24 (3,6,24)	26 (51)	14/12	12.46 ± 2.19 (9–17)	NA	NA	NA
5	Wang et al. (17)	Prospective, one-arm study	China	2015.01–2015.07	24 (1,3,6,12,24)	59 (59)	27/32	12.03 ± 2.31 (7–18)	−3.70 ± 1.39	8 h	YES (by contacting the author)
6	Yan et al. (65)	Prospective, one-arm study	China	2013.1–2013.8	6 (1,6)	59 (59)	30/38	NA (8–14)	NA	NA	NA
7	Yang et al. (18)	Cohort study	China	2018.01–2018.06	12 (1,3,6,12)	60 (60)	23/37	12.6 ± 2.5 (8–14)	−3.50 ± 0.80	8 h	YES
8	Yu-Ling et al. (19)	Cohort study	China	2016.01–2016.06	12 (12)	36 (72)	21/15	13.61 ± 2.41 (8–18)	−1.38 ± 0.23	8–10 h	YES (by contacting the author)
9	Zhang et al. (20)	Prospective, one-arm study	China	2017.01–2017.11	12 (1,3,6,12)	55 (106)	26/29	12.04 ± 2.51 (12–18)	NA	NA	NA
10	Zhao et al. (21)	Randomized controlled trial	China	2019.01–2020.08	12 (3,6,12)	20 (40)	10/10	11.00 ± 1.17 (8–14)	−2.75 ± 0.46	8 h	NA

*NA, not available; SE, spherical equivalent*.

#### Quality Assessment

In the NOS scale, the scores of the nine included articles were >5 stars, suggesting that the quality of the included articles was relatively high. Table 2 makes specific provisions for the score of the comparability column in the NOS scale based on the contents of this study. The first confounder is defined as lens care solution, and the second confounder is defined as sleeping duration. Only when a study reported having a control group and the first and the second confounders were described clearly could two stars be distributed to it, such as the studies by Yang, L. et al. and Yu-Ling, N. et al. Both the studies by Wang, X. et al. and Liu, X.L. et al. rated one star (the former lacked a control group, while the latter lacked adjustment for the second confounder). The remaining articles are ranked 0 in this category. The final score of each included article is shown in Table 3. In addition, since the study of Zhao, Q. et al. was an RCT study, the Jadad scale was used for scoring (see Table 4 for details).

**Table 2:** Quality evaluation of included studies using NOS.

**Study**	**Selection**				**Comparability**	**Outcome**			
	**Representativeness of the Exposed Cohort**	**Selection of the Non-Exposed Cohort**	**Ascertainment of Exposure**	**Demonstration That Outcome of Interest Was Not Present at Start of Study**	**Comparability of Cohorts on the Basis of the Design or Analysis**	**Assessment of Outcome**	**Was Follow-Up Long Enough for Outcomes to Occur**	**Adequacy of Follow Up of Cohorts**	**Total Scores**
Li et al. (13)	⋆	NA	⋆	⋆	0	⋆	⋆	⋆	6
Liu et al. (14)	⋆	⋆	⋆	⋆	⋆	⋆	⋆	⋆	8
Na et al. (15)	⋆	NA	⋆	⋆	0	⋆	⋆	0	5
Shi et al. (16)	⋆	⋆	⋆	⋆	0	⋆	⋆	⋆	7
Wang et al. (17)	⋆	NA	⋆	⋆	⋆	⋆	⋆	⋆	8
Yan et al. (65)	⋆	NA	⋆	⋆	0	⋆	⋆	⋆	6
Yang et al. (18)	⋆	⋆	⋆	⋆	⋆⋆	⋆	⋆	⋆	9
Yu-Ling et al. (19)	⋆	⋆	⋆	⋆	⋆⋆	⋆	⋆	⋆	9
Zhang et al. (20)	⋆	NA	⋆	⋆	0	⋆	⋆	⋆	6

*NOS, Newcastle-Ottawa quality assessment score*.

*⋆The star indicates a score of 1, and ⋆⋆ indicates a score of 2*.

*A follow-up period of equal to or more than 3 months was considered as “follow-up long enough for outcomes to occur”*.

*A star is allocated when the cohort has a loss of follow up rate of <10%*.

**Table 3:** Scoring scale for “Comparability” column in Newcastle-Ottawa Scale.

**Control Group**	**Is the 1^**st**^ confounder adjusted or not?**	**Is the 2^**nd**^ confounder adjusted or not?**	**Score**
√	√	√	⋆⋆
√	×	√	⋆
√	√	×	⋆
√	×	×	0
×	√	√	×
×	×	√	0
×	√	×	0
×	×	×	0

*“√” refers to “Yes”, and “ × ” refers to “No”*.

*⋆The star indicates a score of 1, and ⋆⋆indicates a score of 2*.

*When a study gets three and two “√” in the Table 3 it will be assigned “⋆⋆” and “⋆”, respectively in the “Comparability” column in Table 2. When a study gets zero “√” or three “ × ” in the Table 3 it will be assigned “0” in the “Comparability” column in Table 2*.

*The 1^st^ confounder is defined as the lens care solution. The clear descriptions of the treatments, including lens care solution, cleaning steps and (which) solution used for wetting before wearing is regarded as adjusting this confounder*.

*The 2^nd^ confounder is defined as the sleeping duration. Having the available information of sleeping duration is regarded as adjusting this confounder*.

**Table 4:** Modified Jadad Score for < Clinical efficacy of 0.01% atropine in retarding the progression of myopia in children>.

**Items**	**Score Standard**			**Score**
	**0**	**1**	**2**	
Randomization	Not randomized or inappropriate method of randomization	The study was described as randomized	The method of randomization was described and it was appropriate	1
Concealment of allocation	Not describe the method of allocation concealment	The study was described as using allocation concealment method	The method of allocation concealment was described appropriately	0
Double blinding	Not blind or inappropriate method of blinding	The study was described as double blind	The method of double blinding was described and it was appropriate	0
Withdrawals and dropouts	Not describe the follow-up	A description of withdrawals and dropouts		1
			Total	2

#### Analysis of Heterogeneity

In the heterogeneity test, *p* < 0.01, I^2^ = 95.90% (TBUT, 1 month vs. baseline), p < 0.01, I^2^ = 94.60% (TBUT, 6 months vs. baseline), *p* = 0.07, I^2^ = 54% (Schirmer I test, 6 months vs. baseline), *p* < 0.01, I^2^ = 98.1% (OSDI, 6 months vs. baseline), suggesting that there was a relatively large heterogeneity in each analysis of the included articles (*p* < 0.05 or I^2^ > 50 indicates a relatively strong statistical heterogeneity). Due to the small number of included studies, subgroup analysis was not considered when further analyzing the source of the large heterogeneity. Instead, sensitivity analysis was conducted for the included literature to confirm each difference by sequentially omitting each of the included studies and recalculating the summary difference and 95% CI. *P* value and I^2^ did not decrease significantly after the sequential removal of each article.

#### The Total Effect

**Figure 3:**
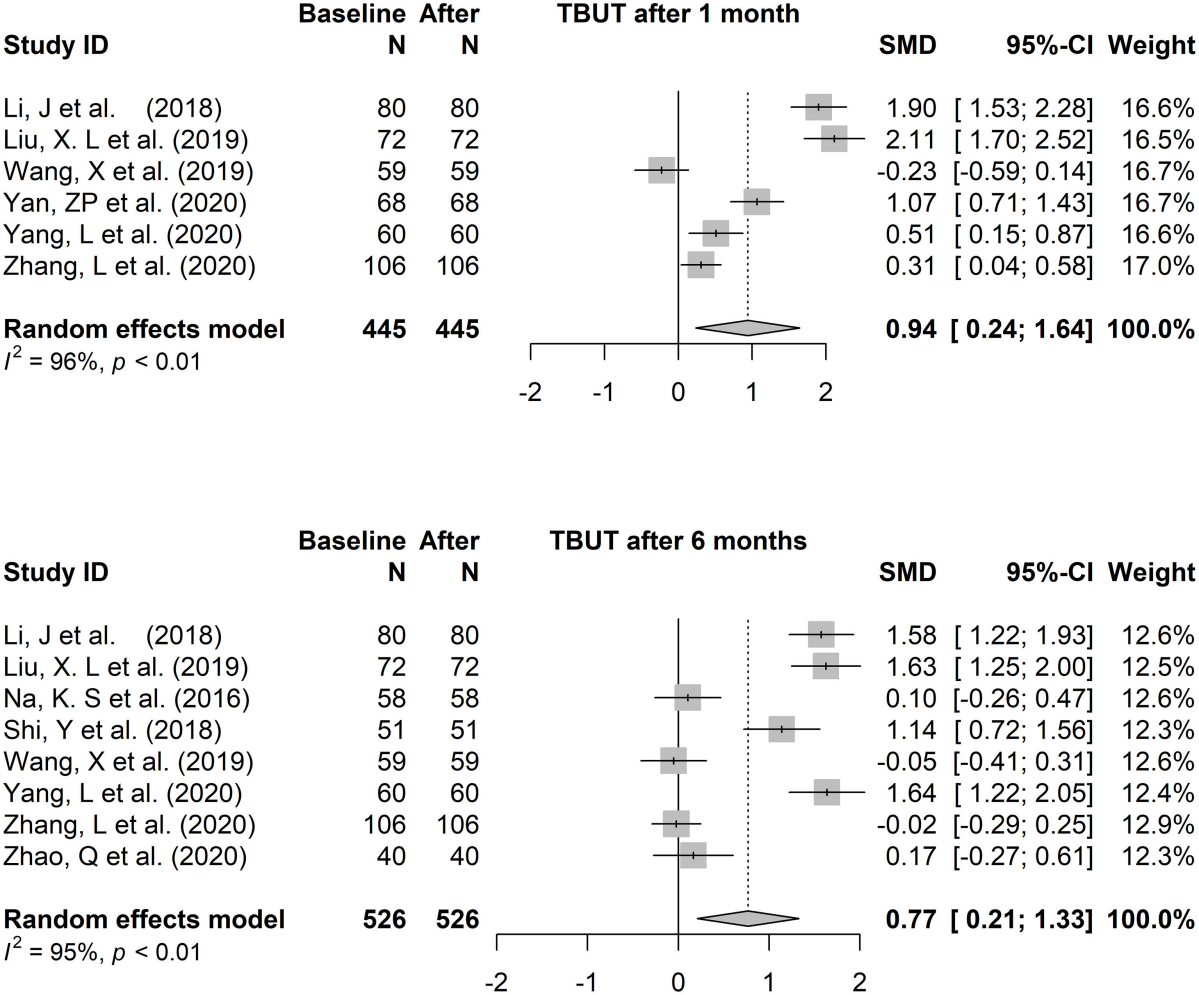
The forest plots of tear break-up time changing after one to 6 months of orthokeratology wearing.

**Figure 4:**
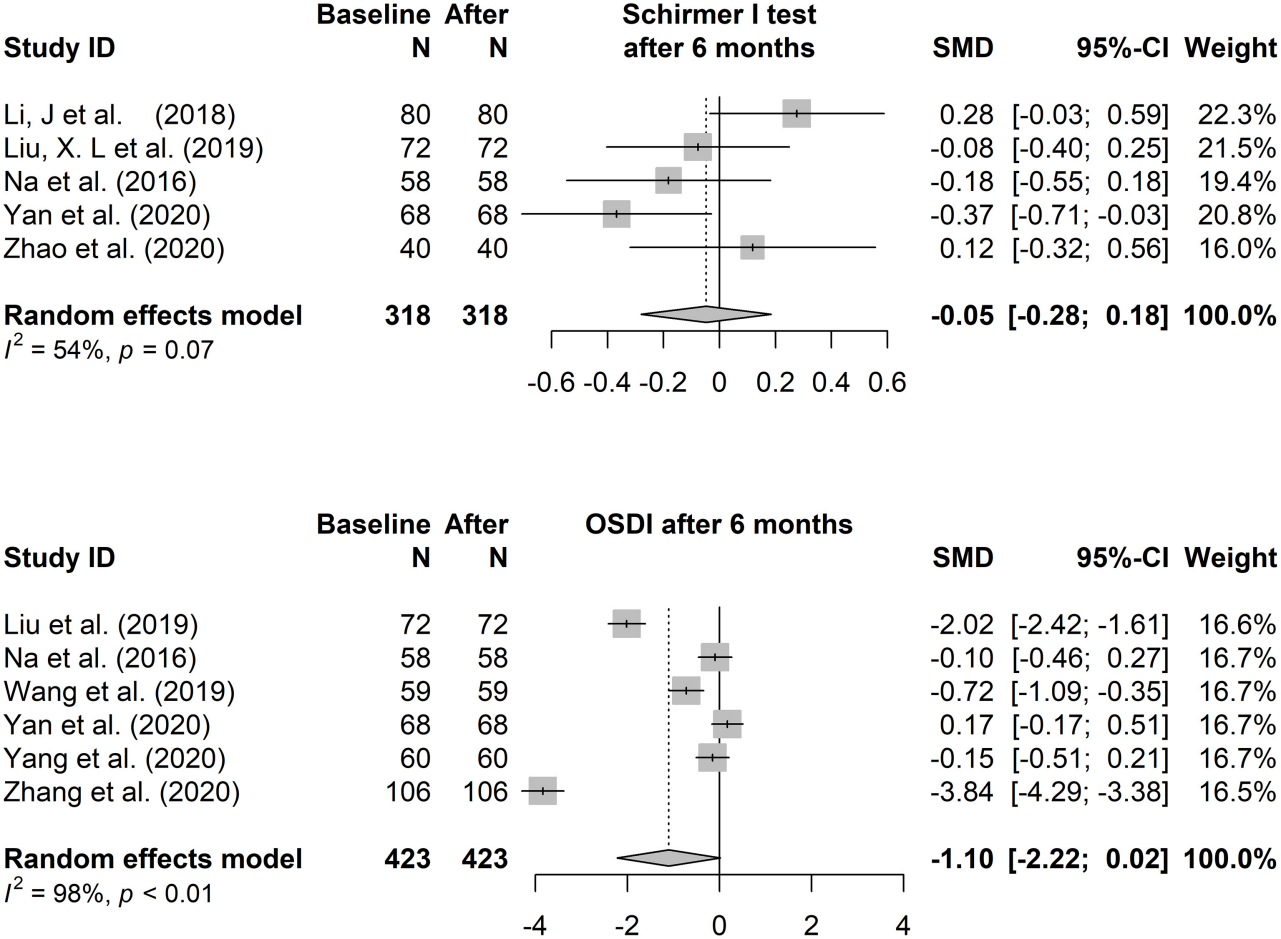
The forest plots of the Schirmer I test and Ocular Surface Disease Index changing after 6 months of orthokeratology wearing.

The fixed-effects model (Hedges' g) and random-effects model (DL) was applied to the combined effect size. The forest plots are shown in Figures 3, 4. The total effect of change in tear film stability before and after orthokeratology wearing was: 0.94 (95% CI: 0.24, 1.64), z = 2.62, *p* = 0.009^*^ (after 1 month), 0.77 (95% CI: 0.21, 1.34), z = 2.69, *p* = 0.007^*^ (after 6 months). Compared with the baseline, the total effect of the change in tear secretion after orthokeratology wearing was: −0.05 (95% CI: −0.28, 0.18), z = −0.56, *p* = 0.577, and the counterpart in OSDI was: −1.10 (95% CI: −2.22, 0.02), z = 1.93, *p* = 0.053.

The forest plots of TBUT change 3 months and 12 months after orthokeratology wearing were shown in Appendices 2A,B. In order to maintain consistency, only the analysis results of 6 months were discussed in this paper.

## The Present Research Dilemma

Although the total effect of TBUT seems to indicate that orthokeratology negatively affects the tear film stability, the conclusions between the included studies showed great different. (a similar situation is also shown in the analysis part of tear secretion and OSDI) and thus resulted in the relatively strong statistical heterogeneity. When the studies of Wang, Zhang Zhao, and Na et al. (denoted as Group A), or the studies of Yang, Li, and Liu et al. (denoted as Group B), were retained, the heterogeneity (the main study of “TBUT after 6 months”) decreased significantly (Group A: *p* = 0.829, I^2^ = 0%; Group B: *p* = 0.986, I^2^ = 0%). The reason for heterogeneity was investigated for the above results. However, in the information provided by all the articles, such as age, diopter, sex ratio, and region, no statistically significant inter-group differences were found between the literature of groups A and B.

This makes it necessary to treat this result with caution and explore possible causes.

### The Differences of the Control Variables

#### The Choice Break-Up Time Test

Different test methods will significantly affect the results of tear film stability. Compared with accessing the data of tear secretion, which has been uniformly tested by the Schirmer I test in relevant studies, tear film stability is usually measured by a tear film BUT test, which has different measurement methods and equipment, mainly including invasive TBUT and non-invasive TBUT. The invasive tear film H test by sodium fluorescein and cobalt blue filter on the slit lamp has been in use for decades and was first proposed by Norn (22). The test is typically performed using a strip of sodium fluorescein moistened with saline or eye lotion to touch the lower eyelid or sclera (or introduce a drop of 1% sodium fluorescein to the lower fornix) and perform the observation with the cobalt blue light in the slit lamp after asking the patient to blink several times. However, the volume of sodium fluorescein introduced to the eye surface is considered an important factor affecting the measurement results of tear film TBUT (23, 24). A non-invasive tear film rupture test is mainly carried out by specially designed tear film measuring devices such as the Keratograph 5M, Oculus Keratograph 4, or Medmont E300 CornealTopographer, which is being widely used (25–27).

The test process of NIBUT does not interfere with the surface of the eye. Therefore, it is more acceptable for children and has good repeatability (28). However, comparing the results of different studies should be treated with caution due to the poor comparability between different equipment (29, 30). TBUT, on the other hand, has been applied clinically for decades, and TBUT (sec) in healthy people is generally less than the NIBUT, even though the minimum measurable value of the latter is lower (24, 31). Fluorescein staining can affect the physiological properties of the tear film, but it can also reflect, to a certain degree, the changes in the tear film stability caused by this external irritant. Compared with NIBUT, the magnifying effect of this traditional test indicates a more sensitive non-significant reduction in tear film quality. Therefore, the data of NIBUT is recommended for presentation in the paper, based on good repeatability and convenience. Also, TBUT should be carried out, and the procedures should be documented as thoroughly as possible.

#### Lens Treatment Before Wearing

The lens treatment before wearing includes disinfection, washing, and lubrication. Because most of the current multipurpose care solutions for disinfection cannot directly contact the eyeball, eye care providers usually require patients to clean the lens before wearing (32, 33). Since the use of distilled water to rinse the lens may change the tear quality during subsequent wearing due to its tendency to change the pH and osmotic pressure of the tear, the use of saline solution, which is close to the physiological osmotic pressure of tears, can help reduce irritation and may be appropriate for lens rinsing. In order to avoid lens-related corneal infections, tap water is not recommended for lens rinsing due to the diverse sterilizing standards of tap water in different countries (34, 35).

In addition, the current research shows that, after cleaning the lens, whether to continue to fill the lens with solution (mainly to increase the wearing comfort) and the type of solution (such as saline solution, artificial tears, etc.) will significantly affect the measurement results of the tear film stability (36–38). The study by Carracedo et al. showed that the corneal staining in the group using sodium hyaluronate (0.3% Ocu-Dry) before wearing glasses was significantly less than the group using saline solution and rewetting drops, but there was no significant difference in TBUT among the three groups (38). However, a recent study by Liu et al. showed the opposite result. The TBUT of the group using sodium hyaluronate (0.1% Hylo) before wearing glasses was higher than the group using saline solution and rewetting drops (37). Carbomer eye drops, another artificial alternative to tears, showed similar results in the study by Shi et al. (36).

Since the processing steps of the lens before wearing may affect the measurement of tear quality, it is advisable to record the lens treatment process, especially the solution used in the washing and lubrication steps.

#### Sleep Duration

Current research has shown a close relationship between sleep, sleep duration, sleep quality, and changes in tears. In a study in Singapore and India, short sleep duration was shown to be significantly and independently associated with dry eye symptoms (39). Similarly, in a study by Zhao et al., which focused on a group of dry eye patients, TBUT and tear secretion were tested in three groups of dry eye patients with different sleep durations. It was revealed that the group with <6 h of sleep duration performed worst in the tests, while the counterparts with 8 h showed the best results (40). In a study by Lee et al., the healthy subjects in the sleep deprivation group performed significantly worse on the TBUT and Schirmer test than the control group with normal sleep (41). Although there is no evidence that tear production and stability of those who sleep more than 6 h vary over time, these studies have only been conducted on adults (39, 40, 42). The relationship between sleep duration and changes in tears has rarely been studied in children and adolescents. Therefore, for future researchers who wish to perform studies on the effects of orthokeratology on the ocular surface in children and adolescents, we recommend that they specify and state the sleep duration in the article.

The American Academy of Pediatrics suggested that children 6 to 12 years of age should sleep 9 to 12 h per 24 h, and 8 to 10 h per 24 h for teenagers between 13 and 18 years of age regularly to promote optimal health (43). Therefore, it may be a more appropriate choice to let the subjects maintain 9–10 h of sleep to further increase the research homogeneity in this field.

#### Data Measuring Time

Sleep itself has a significant effect on tear quality (secretion and stability). When the eyelid closes, inflammatory changes occur in tears in the conjunctival sac, accompanied by neutrophil accumulation, plasminogen activation, increased albumin levels, pro-inflammatory factors, and complement activators (44–46). The tear osmolarity reaches the lowest level for the whole day after waking up, and the ocular surface is rich in inflammatory factors, such as Immunoglobulin A (SIgA) polymorphonuclear leukocytes and complement proteins (47). This change may be associated with corneal physiological edema and “sub-inflammation status,” caused by the closure of the eyelids during sleep and the lack of enough atmospheric oxygen to exchange on the anterior surface of the cornea. In the study of Patel et al., the tear instability reached a roughly balanced state near noon (10 am−12 pm) (48).

In recent years, there have been many studies on how tear quality changes over the course of a day. In a study by Bitton et al., the tear film stability of the dry eye group was at its worst at 7 a.m. and 10 p.m., but the TBUT in the healthy group did not show a significant change over the daytime (49). Lira et al. further studied changes in tear film stability in healthy people over diurnal variation. They found that the tear film stability of healthy people was slightly worse at 5 p.m. than at 9 a.m., which was particularly evident in the test of the tear film stability using fluorescein staining, indicating that the resistance of tears to stimuli was significantly reduced in the evening (50). The previous studies show a rough trend that tear quality is at its worst in the early morning and gradually reaches a more stable state toward noon. The quality of tears decreases slowly over the course of the day and becomes more apparent when sleep deprivation occurs.

On the other hand, compared with the RGP lens, which is worn during the day and in full contact with the cornea (the eye surface will get sufficient tear exchange after each blink), the orthokeratology lens is worn at night, and the lack of blink leads to less tear exchange, which may further increase the “sub-inflammatory” reaction of the eye surface during sleep. Therefore, it is imperative to choose a suitable time to evaluate the tear quality of the OK lens wearer. The ideal timing should be after most of the sleep-induced ocular surface changes have subsided and before the tear quality has declined. Therefore, we suggest that researchers clearly describe the measuring time (period) in their paper. Implying measurements at the time near noon, such as 10 a.m.−12 p.m., may be a benefit to obtain more accurate data and increase homogeneity between studies.

#### Appropriate Types of Study and Quality Assessment Tools

Since the effectiveness of orthokeratology for myopia control has been proven, the essence of this study is about the complications associated with a kind of proven effective intervention for myopia (2, 3). Whether from the perspective of experimental ethics or feasibility, it is difficult for the subject in this paper to implement blinding and randomization in research, especially for the researchers in clinical work. Prospective cohort studies may be the appropriate study design for this subject (51). First of all, a prospective study is about the process from “cause” to “result,” which can better explain whether there is a causal relationship between orthokeratology lens wearing and tear quality change. Secondly, the design of a control group in a cohort study helps analyze and eliminate the influence of unknown interference factors on the results.

In addition, with the emergence of increasing methods for myopia control, such as low-concentration atropine, peripheral defocus glasses, visual perception training, etc., clinical studies in the setting of blank control groups need to consider a larger proportion of ethical factors. Therefore, more clinical studies adopted single-arm studies, which are conducted by investigating the change before and after the main intervention (52). Among the 10 articles included in this study, five were single-arm studies. Single-arm studies often show significant heterogeneity between the results of different studies due to the absence of control groups, as shown in the meta-analysis above. At the same time, due to the lack of appropriate quality assessment tools, this study adopted the NOS scale set for the cohort study. This can hardly be considered suitable for single-armed studies, as can be seen from columns of “Selection of the Non-Exposed Cohort” and “Comparability of Cohorts on the Basis of the Design or Analysis.” Therefore, it is necessary to develop appropriate quality assessment scales for single-arm studies, which are increasingly and commonly conducted in ophthalmology and many other areas, such as oncology or other rare diseases.

#### The Spatial Distribution of the Research Sites

The unbalanced spatial distribution of research is an important aspect that should be highlighted here. The studies by Jennifer et al. showed that the TBUT in Caucasian populations was significantly longer than in Asians in the adult population (53). This conclusion is consistent with previous research (54–56). However, in the study of Kim et al., there was no significant difference in tear film stability between Asian and Caucasian children (aged from 5–18 years) (57). In addition, the study by Wang et al. suggested that differences in tear quality between people were more influenced by the growth environment than by ethnic factors (58). The study by Wang et al. may provide a reasonable explanation for the non-significant difference in tear film stability between the two races in the study by Kim et al.

Whether ethnic factors or geography plays a more important role in the differences in tear film stability in the study, especially among children, is still unclear, which needs to be answered by more multi-center studies with larger samples in the future. However, what is certain is that Asians who grow up in Asia have different ocular surface features than people who grow up in Europe or America. This may be closely related to climatic, sociocultural, genetic, and other factors. This suggests that the comparability of studies conducted in different districts is limited, and it is not advisable to blindly summarize and discuss the studies with various ethnic features and spatial distribution. Secondly, all 10 studies included in this research were in Asian populations. Future studies should fill gaps in this field with European, African, and American populations, which will have profound implications for better understanding the complications of orthokeratology.

#### Usage of Electronic Products

Current research has shown that the usage of electronic products is an important factor in the development of dry eye (59–61). A study on healthy people performed by Bettach et al. showed that the break up time related parameters were significantly reduced after the reading task by smartphone screen (62). The study by Zhu et al. showed that screen time was one of the risk factors for dry eye disease, and different electronic screen time had a significant impact on the change of tear quality (63). In addition, recent studies have shown that electronic products with different screen materials have different effects on the eye surface. Yuan et al. showed that after reading for the same period of time using electronic screens, subjects using eINK screens showed less negative changes on BUT, OSDI and other indicators compared with subjects using OLED screens (64).

It is not difficult to speculate that in the process of orthokeratology wearing, electronic products also have a non-negligible effect on the change of tear quality. However, this factor is often not taken into account in the current studies. Therefore, in future studies, it may be a feasible method to obtain the data of electronic product usage by questionnaires to improve the persuasibility of conclusions.

### Views on the Results of The Meta-Analysis

The results of meta-analysis indicated that orthokeratology had adverse effects on tear film stability. However, in the short term (<6 months), the negative effect is limited in terms of the combined effect size. Except for the study of Yang, L et al., the other included studies with repeated measurements did not show a trend of gradual decrease of BUT over time. Similar results were found for OSDI. However, we found in clinical work that patients with obvious ocular surface discomfort were prone to intermittent wearing or even giving up wearing within a few months. Some subjects with high OSDI scores may be lost to follow-up during the study, which makes it difficult to present the true situation in the results. On the other hand, the Schirmer I test consistently showed no significant change in each study before and after orthokeratology wearing, which would be expected to reflect the fact that orthokeratology had no significant effect on the function of lacrimal glands.

The present results suggested that orthokeratology is more likely to be treated as a safe method of myopia control with less impact on tears. But until more rigorous research comes out, we remain cautious. In addition, compared with OSDI and Schirmer I test, BUT was suitable as the main indicator to study the effect of orthokeratology wearing on tear quality.

## Limitation

There are some weaknesses in this study. First of all, in the meta-analysis section, the primary limitation is that <10 articles are included. Secondly, the large heterogeneity makes the conclusion less convincing, and too few included articles lead to the difficulty of heterogeneity analysis. In addition, it is difficult to include some other common ocular surface indicators, such as tear meniscus height, corneal staining. Finally, in the discussion section, this review was unable to detail the various factors that influence the ocular surface during orthokeratology wearing, such as season factor across the repeated measurements.

## Conclusion

In this study, the results of the meta-analysis suggested that orthokeratology negatively affected tear film stability but had no significant effect on tear secretion or ocular surface symptoms. However, the relatively strong statistical heterogeneity makes it necessary to treat the current results with caution because the results from one study to another showed large differences which is difficult to ignore. Tear quality is affected by sleep duration, lens treatment before wearing and usage of electronic products. Also, the influence of different measurement methods and times on tear film stability should not be ignored and should be carefully chosen by researchers. In the future, more high-quality prospective cohort studies are expected to provide new evidence to explore the complications of orthokeratology. Appropriate quality assessment tools are anticipated to be available for single-arm studies, as they are instructive for research. Finally, the exploration of orthokeratology complications needs to involve more regions and ethnicities to reduce bias.

## Author Contributions

ZT, JW, and MZ conceived the idea and conceptualized the study. ZL and YT collected the data. YT and HD analyzed the data. ZT and JW drafted the manuscript. ZT, JZ, and HD reviewed the manuscript. All authors read and approved the final draft.

## Funding

This study was supported by International cooperative research project (GJHZ20190821113 401670); Sanming Project of Medicine in Shenzhen (SZSM201812090); Supported by Shenzhen Fund for Guangdong Provincial High-level Clinical Key Specialties (No. SZGSP014).

## Conflict of Interest

The authors declare that the research was conducted in the absence of any commercial or financial relationships that could be construed as a potential conflict of interest.

## Publisher's Note

All claims expressed in this article are solely those of the authors and do not necessarily represent those of their affiliated organizations, or those of the publisher, the editors and the reviewers. Any product that may be evaluated in this article, or claim that may be made by its manufacturer, is not guaranteed or endorsed by the publisher.
