# Supplementary material for: Does Orthokeratology Wearing Affect the Tear Quality of Children?
Source: Front Pediatr. 2022 Jan 18;9:773484. doi: 10.3389/fped.2021.773484 (PMC8804288; doi:10.3389/fped.2021.773484)
Supplement: Supplementary file 1 [file Table_5.DOCX]

**Appendix 1**

Pubmed

(((((((((((((Orthokeratologic Procedures[MeSH Terms]) OR (Orthokeratologic Procedure[Title/Abstract])) OR (Procedure, Orthokeratologic[Title/Abstract])) OR (Procedures, Orthokeratologic[Title/Abstract])) OR (ortho-k[Title/Abstract])) OR (OK lens[Title/Abstract])) OR (orthokeratology[Title/Abstract])) OR (Menicon Z Night[Title/Abstract])) OR (overnight contact lens[Title/Abstract])) OR (overnight contact lenses[Title/Abstract])) OR (overnight corneal reshaping[Title/Abstract])) OR (Paragon CRT[Title/Abstract]))) AND (((((((break up time) OR (break-up time)) OR (breakup time)) OR (breaking up time)) OR (NIKBUT)) OR (NITBUT)) OR (TBUT))

Embase：

('orthokeratologic procedure':ab,ti OR 'procedure, orthokeratologic':ab,ti OR 'procedures, orthokeratologic':ab,ti OR 'ortho-k':ab,ti OR 'ok lens':ab,ti OR 'orthokeratology':ab,ti OR 'menicon z night':ab,ti OR 'overnight contact lens':ab,ti OR 'overnight contact lenses':ab,ti OR 'overnight corneal reshaping':ab,ti OR 'paragon crt':ab,ti OR 'orthokeratology lens'/exp) AND ('break up time' OR 'break-up time' OR 'breakup time' OR 'breaking up time' OR 'nikbut' OR 'nitbut' OR 'tbut' OR 'tear break-up time'/exp)

Cochrane library

((orthokeratologic procedure):ti,ab,kw OR (procedure, orthokeratologic):ti,ab,kw OR (procedures, orthokeratologic):ti,ab,kw OR (ortho-k):ti,ab,kw OR (ok lens):ti,ab,kw OR (orthokeratology):ti,ab,kw OR (menicon z night):ti,ab,kw OR (overnight contact lens):ti,ab,kw OR (overnight contact lenses):ti,ab,kw OR (overnight corneal reshaping):ti,ab,kw OR (paragon crt):ti,ab,kw OR (orthokeratology lens):MESH)) AND ((break up time) OR (break-up time) OR (breakup time) OR (breaking up time) OR (rupture time) OR (nibut) OR (nikbut) OR (nitbut) OR (tbut))

China National Knowledge Infrastructure

(篇关摘=(泪膜稳定性) + (泪膜破裂时间) ) AND (篇关摘=(角膜塑形镜) + (OK镜) + (硬质角膜接触镜))

Wanfang Data

摘要:(((泪膜稳定性) or (泪膜破裂时间)) and 摘要:(((角膜塑形镜) or (OK镜) or (硬质角膜接触镜))))

**Appendix2a** The forest plots of tear break-up time changing after twelve months of orthokeratology wearing


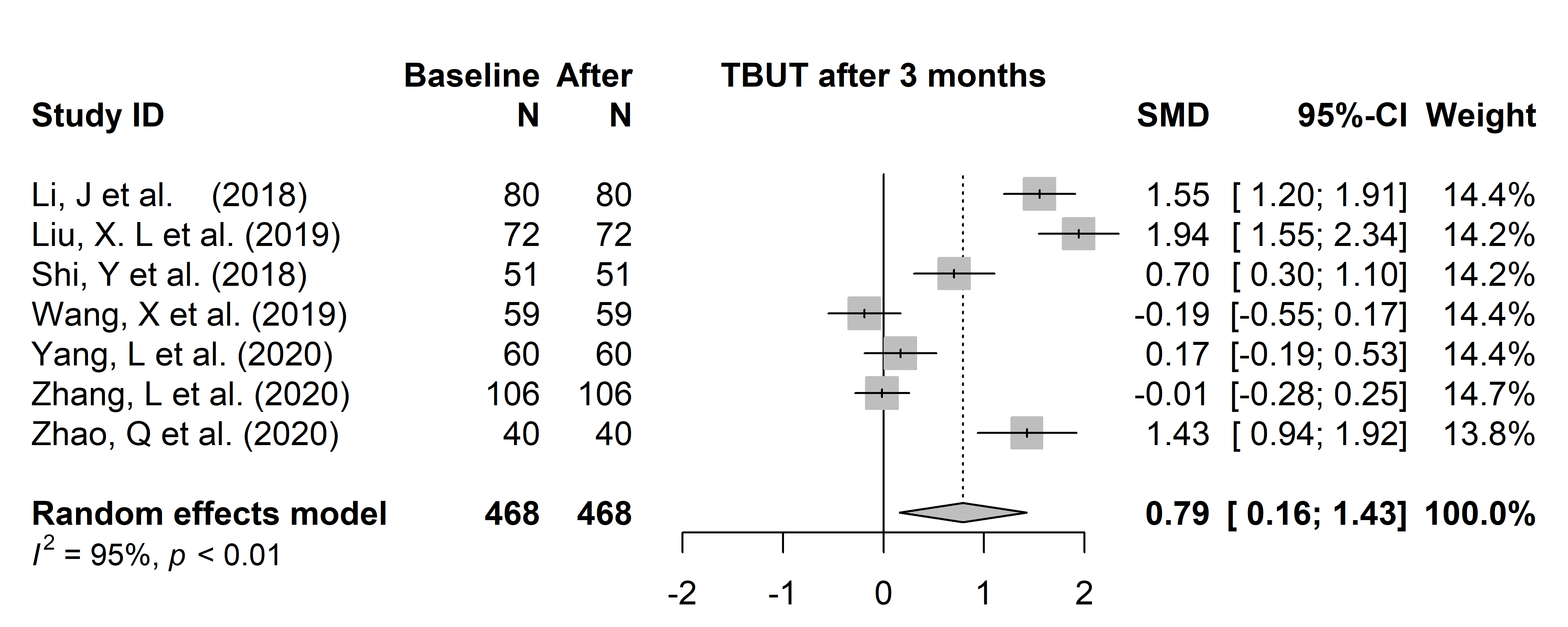


**Appendix2 b** The forest plots of tear break-up time changing after three months of orthokeratology wearing


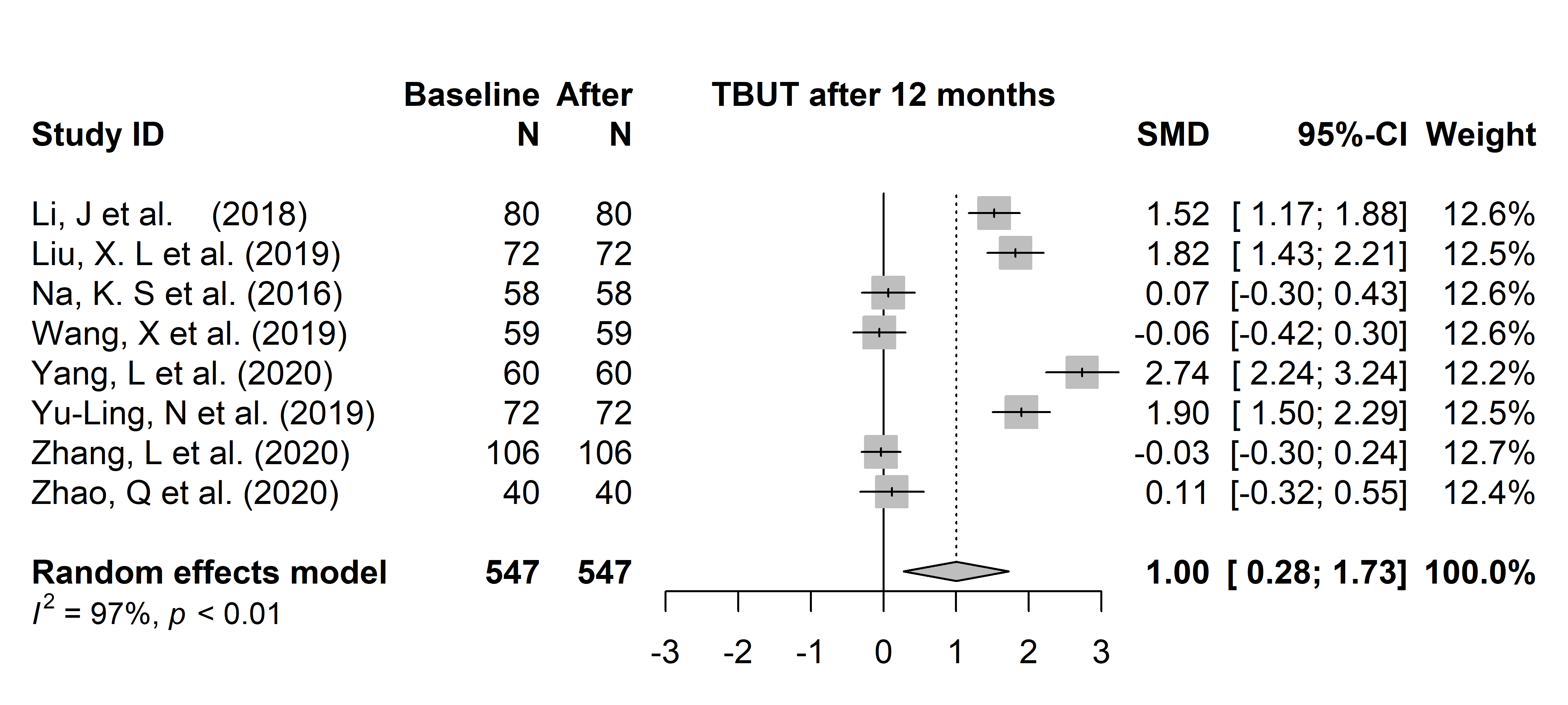


**Appendix3**

Table5a.The OSDI change after 6-month orthokeratology wearing

| ID | Author (Years) | OSDI at baseline | | | Post-orthokeratology 6-month OSDI | | |
| --- | --- | --- | --- | --- | --- | --- | --- |
|  |  | N0 | Mean0 | SD0 | N1 | Mean1 | SD1 |
| 1 | Liu et al. (2019) | 72 | 2.52 | 0.83 | 72 | 6.28 | 2.49 |
| 2 | Na et al. (2016) | 58 | 2.00 | 3.49 | 58 | 2.38 | 4.18 |
| 3 | Wang et al. (2019) | 59 | 4.81 | 6.64 | 59 | 11.40 | 11.01 |
| 4 | Yan et al. (2020) | 68 | 9.80 | 3.24 | 68 | 9.32 | 2.32 |
| 5 | Yang et al. (2020) | 60 | 5.10 | 5.40 | 60 | 6.00 | 6.40 |
| 6 | Zhang et al. (2020) | 106 | 4.80 | 1.63 | 106 | 10.28 | 1.18 |

OSDI: Ocular Surface Disease Index

N0: the number of the subject at baseline; Mean0: the mean value of the OSDI at baseline; SD0: the standard deviation of the OSDI at baseline

N1: the number of the subject after 6-month orthokeratology wearing; Mean1: the mean value of the OSDI after 6-month orthokeratology wearing; SD1: the standard deviation of the OSDI after 6-month orthokeratology wearing

Table5b.The Schirmer I test change after 6-month orthokeratology wearing

| ID | Author (Years) | Schirmer I test at baseline | | | Post-orthokeratology 6-month Schirmer I test | | |
| --- | --- | --- | --- | --- | --- | --- | --- |
|  |  | N0 | Mean0 | SD0 | N1 | Mean1 | SD1 |
| 1 | Li, J et al. (2018) | 80 | 15.60 | 3.31 | 80 | 14.80 | 2.37 |
| 2 | Liu, X. L et al. (2019) | 72 | 14.44 | 2.51 | 72 | 14.64 | 2.64 |
| 3 | Na et al. (2016) | 58 | 20.26 | 9.63 | 58 | 22.10 | 10.43 |
| 4 | Yan et al. (2020) | 68 | 19.34 | 2.55 | 68 | 20.33 | 2.80 |
| 5 | Zhao et al. (2020) | 40 | 18.15 | 1.46 | 40 | 18.00 | 1.01 |

N0: the number of the subject at baseline; Mean0: the mean value of the Schirmer I test at baseline; SD0: the standard deviation of the Schirmer I test at baseline

N1: the number of the subject after 6-month orthokeratology wearing; Mean1: the mean value of the Schirmer I test after 6-month orthokeratology wearing; SD1: the standard deviation of the Schirmer I test after 6-month orthokeratology wearing

Table5c.The TBUT change after 1-month orthokeratology wearing

| ID | Author (Years) | TBUT at baseline | | | Post-orthokeratology 1-month TBUT | | |
| --- | --- | --- | --- | --- | --- | --- | --- |
|  |  | N0 | Mean0 | SD0 | N1 | Mean1 | SD1 |
| 1 | Li, J et al. (2018) | 80 | 10.40 | 2.25 | 80 | 6.60 | 1.69 |
| 2 | Liu, X. L et al. (2019) | 72 | 14.28 | 2.35 | 72 | 9.18 | 2.45 |
| 3 | Wang, X et al. (2019) | 59 | 13.02 | 5.39 | 59 | 14.41 | 6.77 |
| 4 | Yan, ZP et al. (2020) | 68 | 12.30 | 1.82 | 68 | 10.80 | 0.78 |
| 5 | Yang, L et al. (2020) | 60 | 13.50 | 1.90 | 60 | 12.50 | 2.00 |
| 6 | Zhang, L et al. (2020) | 106 | 13.01 | 5.40 | 106 | 11.16 | 6.48 |

TBUT: tear break-up time

N0: the number of the subject at baseline; Mean0: the mean value of the TBUT at baseline; SD0: the standard deviation of the TBUT at baseline

N1: the number of the subject after 1-month orthokeratology wearing; Mean1: the mean value of the TBUT after 1-month orthokeratology wearing; SD1: the standard deviation of the TBUT after 1-month orthokeratology wearing

Table5d.The TBUT change after 6-month orthokeratology wearing

| ID | Author (Years) | TBUT at baseline | | | Post-orthokeratology 6-month TBUT | | |
| --- | --- | --- | --- | --- | --- | --- | --- |
|  |  | N0 | Mean0 | SD0 | N1 | Mean1 | SD1 |
| 1 | Li, J et al. (2018) | 80 | 10.40 | 2.25 | 80 | 7.00 | 2.04 |
| 2 | Liu, X. L et al. (2019) | 72 | 14.28 | 2.35 | 72 | 10.00 | 2.86 |
| 3 | Na, K. S et al. (2016) | 58 | 13.21 | 3.58 | 58 | 12.68 | 6.15 |
| 4 | Shi, Y et al. (2018) | 51 | 9.85 | 1.91 | 51 | 7.81 | 1.63 |
| 5 | Wang, X et al. (2019) | 59 | 13.02 | 5.39 | 59 | 13.31 | 5.81 |
| 6 | Yang, L et al. (2020) | 60 | 13.50 | 1.90 | 60 | 10.20 | 2.10 |
| 8 | Zhang, L et al. (2020) | 106 | 13.01 | 5.40 | 106 | 13.13 | 5.41 |
| 9 | Zhao, Q et al. (2020) | 40 | 12.65 | 1.38 | 40 | 12.40 | 1.59 |

TBUT: tear break-up time

N0: the number of the subject at baseline; Mean0: the mean value of the TBUT at baseline; SD0: the standard deviation of the TBUT at baseline

N1: the number of the subject after 6-month orthokeratology wearing; Mean1: the mean value of the TBUT after 6-month orthokeratology wearing; SD1: the standard deviation of the TBUT after 6-month orthokeratology wearing
