# Supplementary figures and images for: Does Orthokeratology Wearing Affect the Tear Quality of Children?
Source: Front Pediatr. 2022 Jan 18;9:773484. doi: 10.3389/fped.2021.773484 (PMC8804288; doi:10.3389/fped.2021.773484)

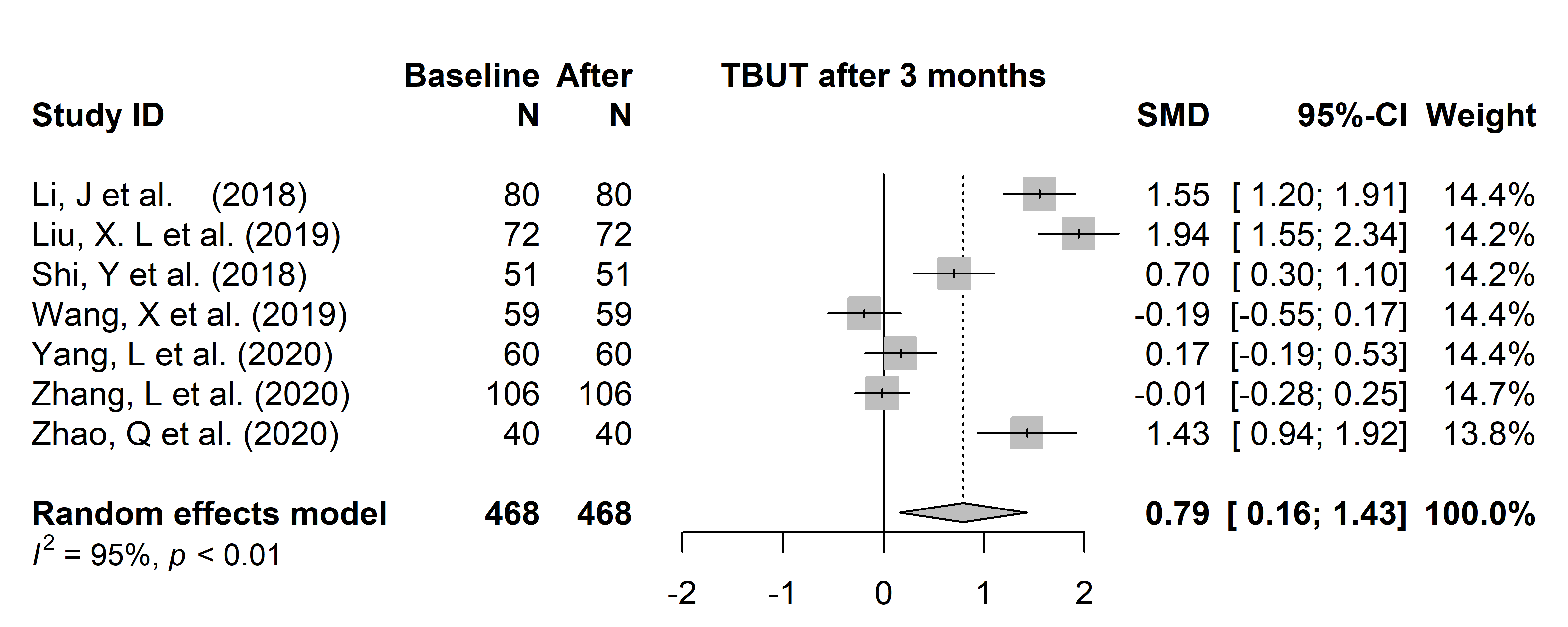

Supplement: Supplementary file 2 [file Image_1.TIFF]

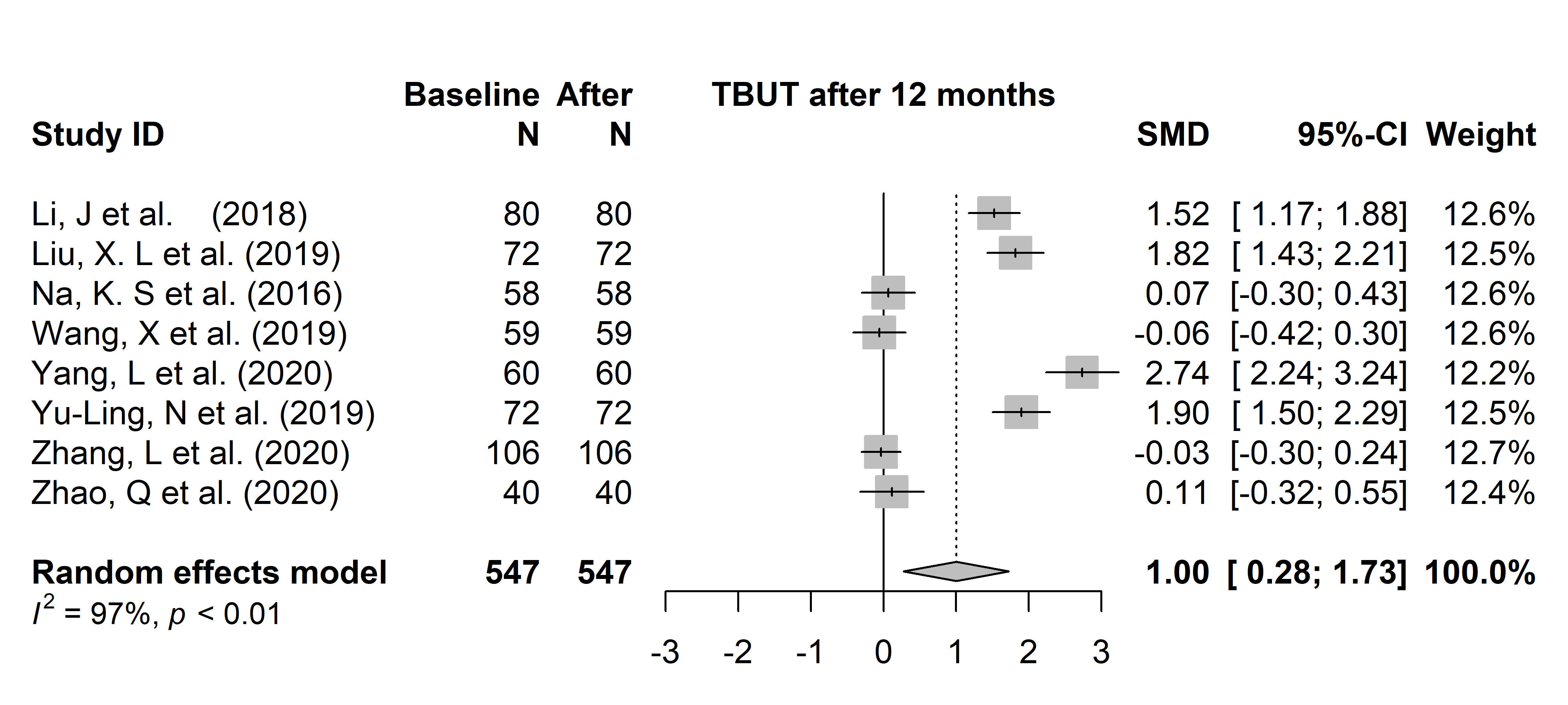

Supplement: Supplementary file 3 [file Image_2.TIFF]
